# A Compensation Method for Nonlinear Vibration of Silicon-Micro Resonant Sensor

**DOI:** 10.3390/s21072545

**Published:** 2021-04-05

**Authors:** Yan Li, Hao Li, Yifeng Xiao, Le Cao, Zhan-She Guo

**Affiliations:** 1 School of Mechanical Electronic & Information Engineering, China University of Mining and Technology-Beijing, Beijing 100083, China; 201572@cumtb.edu.cn (Y.L.); ZQT1900401019G@student.cumtb.edu.cn (H.L.); ZQT2000402056@student.cumtb.edu.cn (Y.X.); 2 School of Electrical and Electronic Engineering, Shanghai University of Engineering Science, Shanghai 201620, China; caole00012@163.com; 3 School of Instrumentation and Optoelectronic Engineering, Beihang University, Beijing 100191, China

**Keywords:** silicon-micro resonant sensor, nonlinear vibration, measurement error, compensation method

## Abstract

A compensation method for nonlinear vibration of a silicon micro resonant sensor is proposed and evaluated to be effective through simulation and experimental analysis. Firstly, the parameter characterization model of the silicon micro resonant sensor is established, which presents significant nonlinearity because of the nonlinear vibration of the resonant beam. A verification circuit is devised to imitate the nonlinear behavior of the model by matching the simulation measurement error of the frequency offset produced by the circuit block with the theoretical counterparts obtained from the model. Secondly, the principle of measurement error compensation is studied, and the compensation method dealing with nonlinear characteristics of the resonant beam is proposed by introducing a compensation beam and corresponding differential operations. The measurement error, compensation rate, and measurement residual between the two scenarios that use single beam and double beams, respectively, are derived and are compared with their simulation and experimental counterparts. The results coincide with the predicted trend, which verifies the effectiveness of the compensation method.

## 1. Introduction

A silicon-micro resonant sensor, as the name implies, is a kind of high-precision sensor that senses the measured object by the change of frequency. It is widely used in civil and military fields owing to its miniaturization, light weight, low energy consumption, high accuracy, good stability, and other advantages [1]. In the field of aerospace, resonant sensors have higher requirements for accuracy. At present, the accuracy of most pressure sensors can only reach 0.075%. The control accuracy directly depends on the measurement accuracy, so the research on its model is the primary task.

The theoretical vibration model is the core formulation to describe the sensitive structure of silicon-micro resonant sensor by using an equivalent single-degree-of-freedom underdamping system. In practical testing, due to the displacement constraint at the support end of the sensitive structure, i.e., no free deformation during vibration, there will be additional internal stress proportional to vibration displacement inside the sensitive structure, causing the natural vibration frequency to vary with different vibration amplitudes of the sensitive structure, thus generating nonlinear vibration. Moreover, with the rapid development of micro-scale silicon-micro machining technology, the vibration amplitude of the micro-scale silicon structure resonant beam is bound to be small when the silicon-micro resonant sensor is sensitive to external input signals, which directly leads to a weak detection signal, limited detection range, and low measurement accuracy. The most direct and effective solution from the source is to increase the amplitude of the resonant beam, enhance the testing signal, broaden the detection range, and improve the measurement accuracy, but large amplitude vibration must inevitably lead to nonlinear vibration. The vibration process of the sensitive structure should ensure the detection range and precision, as well as the vibration stability, so its vibration state should approach the nonlinear vibration, to ensure the detection range and precision of the sensor. Therefore, it is the primary problem to study nonlinear vibration characteristics and corresponding compensation methods for improving the range and accuracy of silicon-micro resonant sensors. 

B. Hok is one of the pioneers who study the nonlinear vibration of medical pressure sensors [2]. Under his leadership, nonlinear studies of various sensors such as photoelectric sensors [3] and magnetic sensors [4] have emerged one after another. Then, the researchers found that by reducing the influence factors of nonlinearity, the measurement accuracy and stability of the sensor can be improved. After a long period of research, it is found that there are many factors affecting the output error of the sensor, such as residual stress [5], electrostatic force [6], large amplitude [7], bias voltage [8], material [9], additional mass [10], and coupling effect [11,12]. For a specific sensor, the performance depends on a variety of factors, instead of considering one single aspect. In order to reduce the influence of different factors on the sensor nonlinear vibration, it is necessary to incorporate it into the nonlinear vibration model, then quantify and solve. Therefore, in the next study, there are many studies on the solution methods, but the dominant ones are the harmonic balance method [13], multiscale method [14], Chebyshev polynomials [15], and KBM method [16]. With the help of these methods, the influence of the nonlinear vibration can be seen directly by analyzing the dynamic performance with stability lobe graphs [17] or measurement experiments [18]. 

Then, through algorithms compensation, the accuracy of the sensor can be improved. For example, D. G. Kim employed the Takagi Sugeno fuzzy model to subtract the estimated output from the original data of the resonant sensor to compensate for the nonlinear deviation drift [19]. C. Pramanik proposed an intelligent online temperature compensation scheme based on artificial neural network technology for a porous silicon micromechanical resistance pressure sensor [20]. G. Araghi introduced a temperature compensation model for a total inertial measurement unit based on a radial basis function neural network, which was used to compensate the measurement error of the accelerometer and three-axis gyroscope [21]. T. Wu designed a surface fitting compensation algorithm based on the least square method, which can effectively eliminate the zero offset and nonlinear error of the silicon piezoresistive pressure sensor [22]. However, with the progress of science and technology, the accuracy of the existing sensors is not enough to meet the demand. The algorithm’s compensation of sensors has stepped onto the development bottleneck.

The above studies mainly focused on the influencing factors, characterization, solutions, and error compensation of the nonlinear vibration of sensors. Through the optimization of each component in the sensor, the performance of the sensor is continuously improved to meet various needs. However, in the study of nonlinear vibration analysis and error compensation, the predecessors mainly established a temperature compensation model through different algorithms to realize the suppression of sensor zero drift and sensitivity drift. In this paper, besides incorporating temperature and axial stress into the dynamic model of resonant beam, we also compensated the measurement error caused by nonlinear vibration.

In our previous work, Li Yan et al. have thoroughly studied the parameter characterization and solution of gyroscope in the resonant state [23] and incomplete resonant state [24], and the frequency measurement method of the gyroscope [25]. All the previous works not only broaden the linear theoretical system of the silicon-micro resonant sensor, but also have a guiding significance for the nonlinear theoretical derivation of this paper.

In this paper, an electrothermal excitation silicon-micro resonant pressure sensor is taken for example. Firstly, the working principle is analyzed theoretically, and the nonlinear vibration parameters characterization of the resonator is established and solved. Then, the measurement error model is developed, the relationship between measurement error and nonlinear vibration is further proven by using circuit blocks, and the compensation method is raised for the nonlinear vibration caused by the electrothermal excitation and measured pressure. Finally, a series of experiments are carried out to verify the feasibility of the compensation method.

## 2. Working Principle of Silicon-Micro Resonant Sensor 

**Figure 1:**
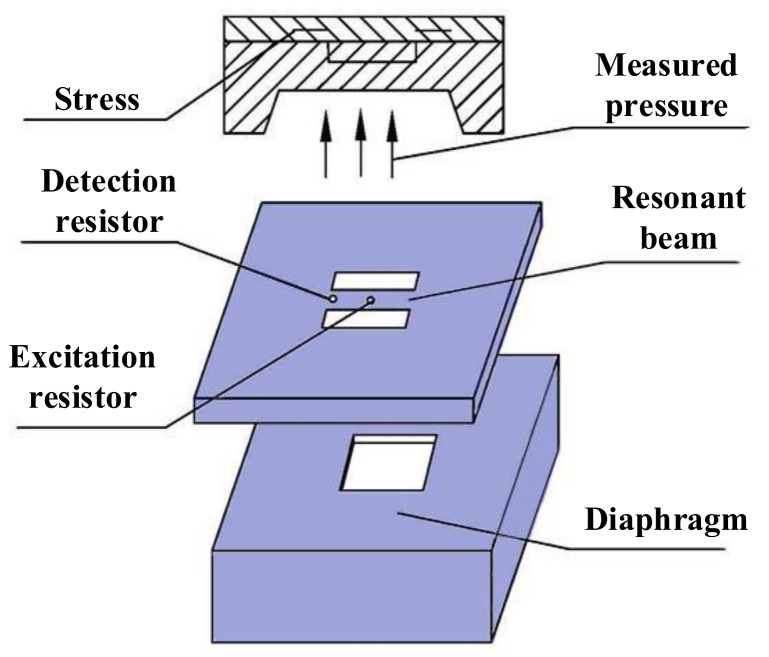
Schematic diagram of the silicon-micro resonant pressure sensor.

The simplified model of the silicon-micro resonant pressure sensor is shown in Figure 1. It mainly includes the sensitive structure, drive unit, detection unit, and closed-loop control unit. The inner part of the resonator will produce axial stress due to the diaphragm deformation under the action of measured pressure, when the sensitive structure that is composed of a square diaphragm and resonant beam is sensitive to the measured pressure, which leads to the shift in the natural frequency. The drive unit excites the resonant beam to vibrate and keeps it in a resonant state. The detection unit picks up the vibration signal of the resonant beam and feeds it back to the drive unit through the closed-loop control unit. Therefore, the frequency of the excitation force is always consistent with the natural frequency of the resonant beam, which realizes tracking of the natural frequency of the resonator. The frequency shift can be obtained by picking up the resonant beam vibration signal, and then calculating the measured pressure. 

As a result of the displacement constraint at the support end of the resonant beam, it cannot be freely deformed when it vibrates, which results in additional internal stress that is proportional to the vibration displacement inside the resonant beam. The existence of internal stress makes the natural frequency of the resonant beam change with the different vibration amplitude, and then produces nonlinear vibrations. For a resonant beam with silicon micro-machining technology, the nonlinear vibration caused by large amplitude is more significant due to its miniaturization in size.

## 3. Measurement Error of the Resonant Beam Nonlinear Vibration

### 3.1. Parameter Characterization of Nonlinear Vibration 

**Figure 2:**
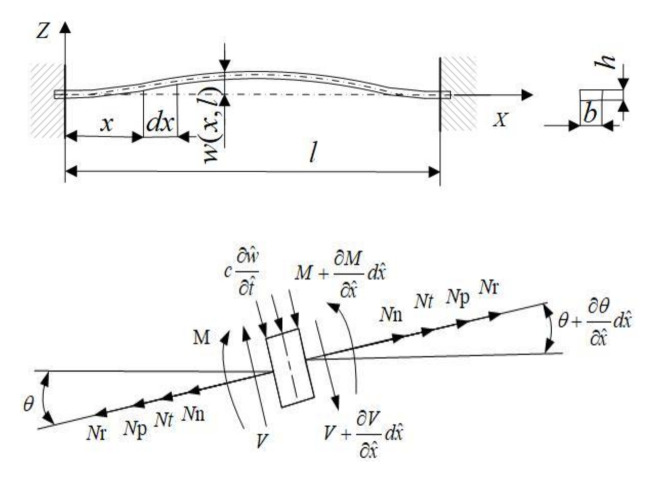
Schematic diagram of micro element stress for the resonant beam.

According to the Euler Bernoulli beam model, the resonant beam of the silicon-micro resonant sensor is simplified. Take any element with length dx along the length of the resonant beam for force analysis, as shown in Figure 2.

Table 1 lists the geometric, elastic, and thermal parameters of the resonant beam and diaphragm involved in the model.

where M is the sum of bending moments $M_{b}(\hat{x},\hat{t})$ and $M_{t}(\hat{x},\hat{t})$, which are caused by the bending deformation of the resonant beam and its temperature gradient along the thickness direction, respectively. They can be expressed as [26].

(1) $$M_{b}(\hat{x},\hat{t})=EJ\frac{\partial^{2}\hat{w}(\hat{x},\hat{t})}{\partial {\hat{x}}^{2}}$$

(2) $$M_{t}(\hat{x},\hat{t})=M_{th1}(\hat{x},\hat{t})+M_{th2}(\hat{x},\hat{t})$$

where $M_{th1}(\hat{x},\hat{t})$ and $M_{th2}(\hat{x},\hat{t})$ are distributed time-dependent driving moments developed, respectively, by the dynamic thermal components of $P_{1}(\hat{t})$ and $P_{2}(\hat{t})$. They can also be expressed as [26].

(3) $$P_{1}(\hat{t})=\frac{2U_{dc}U_{ac}\cos(\hat{\omega }\hat{t})}{R}$$

(4) $$P_{2}(\hat{t})=\frac{0.5U_{ac}^{2}\cos(2\hat{\omega }\hat{t})}{R}$$

where $U_{dc}$ is the dc bias. R is the excitation resistor. $U_{ac}$ and $\overset{⌢}{\omega }$ are the amplitude and frequency of the ac exciting signal, respectively.

Stress analysis of the resonant beam element in vertical direction:

(5) $$\begin{array}{l} \rho A\frac{\partial^{2}\hat{w}}{\partial {\hat{t}}^{2}}d\hat{x}+(V+\frac{\partial V}{\partial \hat{x}}d\hat{x})\cos(\theta +\frac{\partial \theta }{\partial \hat{x}}d\hat{x})-V\cos\theta +c\frac{\partial \hat{w}}{\partial \hat{t}}d\hat{x}\cos\theta \\ +(N_{n}+N_{t}+N_{p}+N_{r})\sin\theta -(N_{n}+N_{t}+N_{p}+N_{r})\sin(\theta +\frac{\partial \theta }{\partial \hat{x}}d\hat{x})=0 \end{array}$$

Moment balance of the resonant beam element:

(6) $$Vd\hat{x}+M-c\frac{\partial \hat{w}}{\partial \hat{t}}d\hat{x}\cdot \frac{d\hat{x}}{2}-(M+\frac{\partial M}{\partial \hat{x}}d\hat{x})=0$$

when θ is small, there $\cos\theta \approx \cos(\theta +(\partial \theta /\partial \hat{x})d\hat{x})\approx 1$.

Substituting the above formula into (5) and combining with (6), and neglecting the high order small quantity, we can get:

(7) $$\rho A\frac{\partial^{2}\hat{w}}{\partial {\hat{t}}^{2}}+\frac{\partial V}{\partial \hat{x}}+c\frac{\partial \hat{w}}{\partial \hat{t}}-(N_{n}+N_{t}+N_{p}+N_{r})\frac{\partial^{2}\hat{w}}{\partial {\hat{x}}^{2}}=0$$

(8) $$V=\frac{\partial M}{\partial \hat{x}}$$

The nonlinear vibration parameters of the electrothermal excited resonant beam are characterized as:

(9) $$\begin{array}{l} EJ\frac{\partial^{4}\hat{w}}{\partial {\hat{x}}^{4}}+\rho bh\frac{\partial^{2}\hat{w}}{\partial {\hat{t}}^{2}}+c\frac{\partial \hat{w}}{\partial \hat{t}}=\left[\frac{Ebh}{2l}\int_{0}^{1}{\left(\frac{\partial \hat{w}}{\partial \hat{x}}\right)}^{2}d\hat{x}+N_{t}+N_{p}+N_{r}\right]\frac{\partial^{2}\hat{w}}{\partial {\hat{x}}^{2}}\\ -\frac{\partial^{2}M_{th1}(\hat{x},\hat{t})}{\partial {\hat{x}}^{2}}-\frac{\partial^{2}M_{th2}(\hat{x},\hat{t})}{\partial {\hat{x}}^{2}} \end{array}$$

The boundary conditions are:

(10) $$\hat{w}(0,\hat{t})=\hat{w}(l,\hat{t})=0$$

(11) $$\frac{\partial \hat{w}(0,\hat{t})}{\partial \hat{x}}=\frac{\partial \hat{w}(l,\hat{t})}{\partial \hat{x}}=0$$

### 3.2. The Solution of the Nonlinear Vibration Parameters Characterization 

Since such formulation will facilitate the identification of the order of magnitude of some variables. Hence let:

(12) $$w=\frac{\hat{w}}{r},\;x=\frac{\hat{x}}{l},\;t={\hat{\omega }}_{1}\hat{t},\;\omega =\frac{\hat{\omega }}{{\hat{\omega }}_{1}}$$

where $r={\left(J/bh\right)}^{1/2}$ is the radius of gyro radius of cross section, ${\hat{\omega }}_{1}$ is the fundamental natural frequency of the resonant beam under linear vibration.

(13) $${\hat{\omega }}_{1}=\frac{4.73}{l^{2}}\sqrt{\frac{EJ}{\rho bh}}\sqrt{1+\frac{0.295(N_{t}+N_{p}+N_{r})l^{2}}{12EJ}}$$

In non-dimensional form, (9), (10) and (11) become:

(14) $$\begin{array}{l} \frac{EJ}{l^{4}}\frac{\partial^{4}w}{\partial x^{4}}+\rho bhr{\hat{\omega }}_{1}^{2}\frac{\partial^{2}w}{\partial t^{2}}+cr{\hat{\omega }}_{1}^{2}\frac{\partial w}{\partial t}\\ =\frac{r}{l^{2}}\left[\frac{Ebhr^{2}}{2l^{2}}\int_{0}^{1}{\left(\frac{\partial w}{\partial x}\right)}^{2}dx+N_{t}+N_{p}+N_{r}\right]\frac{\partial^{2}w}{\partial x^{2}}\\ -\left[\delta_{-1}(\frac{2xl-l+l_{R}}{2})-\delta_{-1}(\frac{2xl-l-l_{R}}{2})\right]\left[M_{d1}(t)+M_{d2}(t)\right] \end{array}$$

(15) w(0,t)=w(l,t)=0

(16) $$\frac{\partial w(0,t)}{\partial x}=\frac{\partial w(l,t)}{\partial x}=0$$

where

(17) $$M_{d1}(t)=\frac{24U_{ac}U_{dc}EJ\beta M(\hat{\omega })}{\lambda Rbl_{R}}\cos\left[\omega t-\phi_{d}(\hat{\omega })\right]$$

(18) $$M_{d2}(t)=\frac{6U_{ac}^{2}EJ\beta M(2\hat{\omega })}{\lambda Rbl_{R}}\cos\left[2\omega t-\phi_{d}(2\hat{\omega })\right]$$

where

(19) $$M(\hat{\omega })=\left|\frac{i}{2\gamma^{2}}\right.\left.\left[\frac{1}{2}+\frac{1}{\gamma (1+i)}-\frac{2}{\gamma (1+i)\left[1+\exp(-\gamma (1+i))\right]}\right]\right|$$

(20) $$\phi_{d}(\hat{\omega })=-\arctan\frac{1-\gamma -(1+\gamma )e^{-2\gamma }-2e^{-\gamma }\left[\gamma \cos\gamma -\sin\gamma \right]}{1-e^{-2\gamma }-2e^{-\gamma }\sin\gamma }$$

where $l_{R}$ is the length of exciting resistance, $\delta_{-1}(x)$ is the first derivative of Dirac function, and λ is the thermal conductivity of monocrystalline silicon. Moreover, where $\gamma =h/\delta ,\;\delta =(2\lambda /\rho c\hat{\omega }{)}^{1/2}$.

It is assumed that the vibration displacement of the nonlinear vibration parameter expression of the resonant beam is expanded according to the main mode as follows:

(21) $$w(x,t)=\sum_{i=1}^{n}u_{i}(t)\varphi_{i}(x)$$

where $\varphi_{i}(x)$ is the *i*-th linear undamped mode function, and satisfies the following relationship:

(22) $$EJ\varphi_{i}^{iv}(x)=\rho bhl^{4}\omega_{i}^{2}\varphi_{i}(x)+(N_{t}+N_{p}+N_{r})l^{2}\varphi_{i}^{\prime \prime }(x)$$

(23) $$\varphi_{i}=0\;\mathrm{and}\;\varphi_{i}^{\prime }=0\;\mathrm{at}\;x=0\;\mathrm{and}\;x=1$$

Since the frequency $\hat{\omega }$ of the driving moment $M_{th1}(\hat{x},\hat{t})$ is set approximately to the fundamental natural frequency ${\hat{\omega }}_{1}$ of the resonant beam by the PLL circuit, it is reasonable to assume that the fundamental mode should be dominant in the vibration. Hence, let n = 1 in (21), and then a reduced order model of the resonant beam is obtained:

(24) $$\begin{array}{l} \;{\ddot{u}}_{1}(t)+\frac{1}{Q_{1}}{\dot{u}}_{1}(t)+u_{1}(t)+\varepsilon u_{1}^{3}(t)\\ =F_{1}(\hat{\omega })cos\left[\omega t-\phi_{d}(\hat{\omega })\right]+F_{2}(2\hat{\omega })cos\left[2\omega t-\phi_{d}(2\hat{\omega })\right] \end{array}$$

where $F_{1}(\hat{\omega })$ and $F_{2}(2\hat{\omega })$ are the amplitudes of the two excitation forces, respectively. The reason why the second-order mode component is introduced as the excitation signal, which is to compensate for the part of energy lost by the nonlinear influence. $Q_{1}$ and ε can be presented as [26]:

(25) $$Q_{1}=\frac{\rho bh{\hat{\omega }}_{1}}{c}\;\varepsilon =\frac{\zeta Er^{2}}{2\rho {\hat{\omega }}_{1}^{2}l^{4}}=0.1098$$

Typically, ε is small (ε=0.1098, when ${\hat{\omega }}_{1}=2\pi \times 40,000\mathrm{rad}/s$). Thus, ε can be used as the small perturbation parameter in the subsequent perturbation analysis. $Q_{1}$ is the quality factor of the resonant beam for the fundamental mode. Noted that since normal operation is under vacuum, $1/Q_{1}<<1$ can be expressed in terms of the ε:

(26) $$1/Q_{1}=2\varepsilon \mu$$

where

(27) $$\mu =\frac{c{\hat{\omega }}_{1}l^{4}}{\xi Ebhr^{2}}$$

The coefficient ξ in (27) is a constant and can be calculated as [26].

(28) $$\xi =-\frac{\int_{0}^{1}\varphi_{1}^{\prime \prime }(x)\varphi_{1}(x)dx\int_{0}^{1}\varphi_{1}^{\prime }{}^{2}(x)dx}{\int_{0}^{1}\varphi_{1}^{2}(x)dx}\approx 60.0087$$

In order to quantitatively describe the proximity between the excitation frequency and the natural vibration frequency of the resonant beam, a detuning parameter σ is introduced, as is given by:

(29) ω=1+εσ

In addition, set $F_{1}(\hat{\omega })=\varepsilon K_{1}(\hat{\omega })$ and $F_{2}(2\hat{\omega })=\varepsilon K_{2}(2\hat{\omega })$,

where

(30) $$K_{1}(\omega )=\frac{48U_{ac}U_{dc}J\alpha_{L}l\varphi_{1}^{\prime \prime }(x_{0})}{\lambda Rb^{2}hr^{3}\int_{0}^{1}\varphi_{1}^{\prime \prime }(x_{0})\varphi_{1}(x)dx\int_{0}^{1}\varphi_{1}^{\prime }{}^{2}(x)dx}M(\hat{\omega })$$

(31) $$K_{2}(2\omega )=\frac{12U_{ac}^{2}J\alpha_{L}l\varphi_{1}^{\prime \prime }(x_{0})}{\lambda Rb^{2}hr^{3}\int_{0}^{1}\varphi_{1}^{\prime \prime }(x_{0})\varphi_{1}(x)dx\int_{0}^{1}\varphi_{1}^{\prime }{}^{2}(x)dx}M(2\hat{\omega })$$

Therefore, $F_{1}(\hat{\omega })$ and $F_{2}(2\hat{\omega })$ can be expressed as:

(32) $$F_{1}(\hat{\omega })=\varepsilon \left[K_{1}({\hat{\omega }}_{1})+\varepsilon K_{1}^{\prime }({\hat{\omega }}_{1}){\hat{\omega }}_{1}\sigma +o(\varepsilon^{2})\right]$$

(33) $$F_{2}(2\hat{\omega })=\varepsilon \left[K_{2}(2{\hat{\omega }}_{1})+2\varepsilon K_{2}^{\prime }(2{\hat{\omega }}_{1}){\hat{\omega }}_{1}\sigma +o(\varepsilon^{3})\right]$$

Substituting (26), (29), (32), and (33) into (24) yields:

(34)  u¨1(t)+2εμu˙1(t)+u1(t)+εu13(t)=εK1(ω^)1+ε2K1′(ω^)1ω^σ1+o(ε3) ×cos2(1+εσ)t−ϕd(ω^)+εK2(2ω^)1+2ε2K2′(2ω^)1ω^σ1+o(ε3)×cos2(1+εσ)t−ϕd(2ω^)

Using the multi-scale method of perturbation method to solve Equation (34), the amplitude frequency response and phase frequency response of nonlinear vibration for resonant beam can be obtained as follows:

(35) $$\hat{\omega }={\hat{\omega }}_{1}+\frac{3{\hat{a}}^{2}}{8r^{2}}{\hat{\omega }}_{1}\varepsilon \pm {\left[\frac{K_{1}^{2}({\hat{\omega }}_{1})r^{2}}{4{\hat{a}}^{2}}-\mu^{2}\right]}^{0.5}{\hat{\omega }}_{1}\varepsilon$$

(36) $$\hat{\omega }={\hat{\omega }}_{1}+\frac{3\varepsilon {\hat{\omega }}_{1}K_{1}^{2}({\hat{\omega }}_{1})}{32\mu^{2}}\sin^{2}\left[\phi -\phi_{d}(\hat{\omega })\right]-\mu \varepsilon {\hat{\omega }}_{1}\cot\left[\phi -\phi_{d}(\hat{\omega })\right]$$

### 3.3. Measurement Error Caused by Nonlinear Vibration

In the phase-locked closed-loop test system of the silicon-micro resonant sensor, the phase-locked loop utilizes the frequency response characteristics to track and lock the natural frequency of the sensitive structure. When the resonant beam vibrates in the linear state, the difference between the excitation and the detection signal is π/2, and the natural frequency can track the resonant beam vibrates at its natural frequency. When the resonant beam vibrates in the nonlinear state, the resonant beam will bend the frequency response characteristic curve, making it difficult for the phase-locked loop to accurately lock the natural frequency, resulting in the measurement error of the sensor. The vibration displacement of the resonant beam is defined as ϕ=π/2, and then the vibration frequency of the resonant beam is obtained as follows:

(37) $$\hat{\omega }={\hat{\omega }}_{1}+\frac{3\varepsilon {\hat{\omega }}_{1}K_{1}^{2}({\hat{\omega }}_{1})}{32\mu }\cos^{2}\left[\phi_{d}(\hat{\omega })\right]-\frac{\tan\left[\phi_{d}(\hat{\omega })\right]}{2Q_{1}}{\hat{\omega }}_{1}$$

The frequency output error can be written as:

(38) $$\frac{\hat{\omega }-{\hat{\omega }}_{1}}{{\hat{\omega }}_{1}}=E_{1}+E_{2}$$

where $E_{1}=(3/8)\varepsilon^{3}Q_{1}^{2}K_{1}^{2}({\hat{\omega }}_{1})\cos^{2}\left[\phi_{d}(\hat{\omega })\right]$ and $E_{2}=-\tan[(\phi_{d}(\overset{⌢}{\omega }))$$/2Q_{1}]$ are errors caused by the resonant beam nonlinear vibration and the phase shift of the electrothermal excitation, respectively. Due to the phase shift of the electrothermal excitation $\phi_{d}(\hat{\omega })\approx 0$, there are:

(39) $$E_{1}\approx \frac{3}{8}\varepsilon^{3}Q_{1}^{2}K_{1}^{2}({\hat{\omega }}_{1})$$

The resonant beam amplitude $\hat{a}$(ϕ=π/2) is:

(40) $$\hat{a}=\frac{K_{1}({\hat{\omega }}_{1})r}{2\mu }$$

Substituting (26) and (30) into (40) leads to:

(41) $$E_{1}=\varepsilon \frac{3}{8}{\left(\frac{\hat{a}}{r}\right)}^{2}$$

Obviously, the measurement error of the resonant sensor caused by nonlinear vibration is not only related to the resonant beam amplitude, but also affected by the coefficient of nonlinear vibration.

### 3.4. Circuit Verification of Nonlinear Vibration Measurement Error

The nonlinear vibration of the silicon-micro resonant sensor will shift the natural frequency of the resonant beam, and then produce measurement errors. Based on the vibration theory and electro-mechanical equivalent principle of the silicon-micro resonant pressure sensor, this section introduces the construction of the equivalent circuit block, which imitates the mentioned nonlinear vibration parameter characterization. The frequency characteristics of the equivalent circuit output waveform are analyzed qualitatively to verify the influence of nonlinear vibration on the measurement error.

Before designing the circuit for characterizing nonlinear vibration parameters, Equation (24) needs to be rewritten to Equation (42) as follows:

(42) $$\begin{array}{l} {\ddot{u}}_{1}(t)=-\frac{1}{Q_{1}}{\dot{u}}_{1}(t)-u_{1}(t)-\varepsilon u_{1}^{3}(t)\\ +F_{1}(\hat{\omega })\cos\left[\omega t-\phi_{d}(\hat{\omega })\right]\;+F_{2}(2\hat{\omega })\cos\left[2\omega t-\phi_{d}(2\hat{\omega })\right] \end{array}$$

In (42), $u_{1}(t)$ can be obtained by two integral calculators. $-(1/Q_{1}){\dot{u}}_{1}(t)$ can be achieved by integrating ${\ddot{u}}_{1}(t)$ and scaling up. $-u_{1}(t)$ can be connected directly to the circuit as a feedback signal. $-\varepsilon u_{1}^{3}(t)$ can be realized by the multiplier and proportional amplifier. The verification circuit works by self-oscillation; $F_{1}(\hat{\omega })\cos\left[\omega t-\phi_{d}(\hat{\omega })\right]$ and $F_{2}(2\hat{\omega })\cos\left[2\omega t-\phi_{d}(2\hat{\omega })\right]$ are used as excitation signals only to make the resonant beam vibrate at its natural frequency, and therefore are not considered. The equivalent verification circuit block diagram of the silicon-micro resonant pressure sensor can be obtained by adding each term involved in Equation (42).

**Figure 3:**
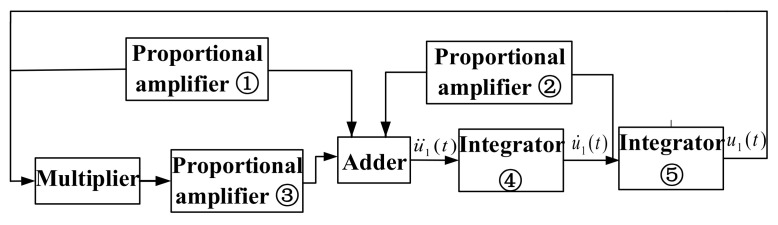
Equivalent verification circuit block diagram of the silicon-micro resonant pressure sensor.

**Figure 4:**
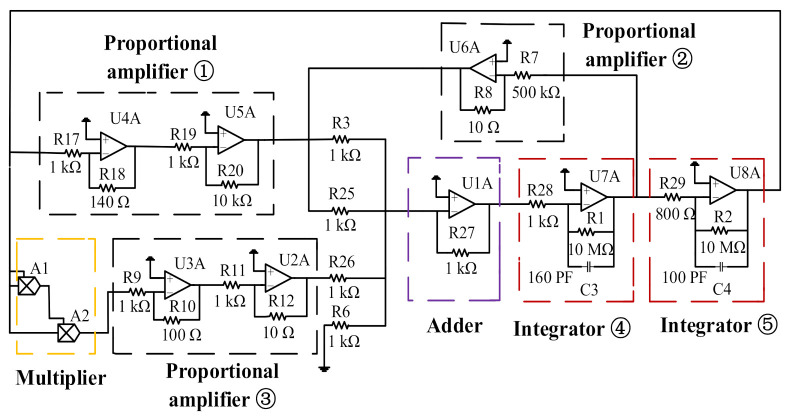
Equivalent verification circuit diagram of the silicon-micro resonant pressure sensor.

As shown in Figure 3, the equivalent verification circuit block diagram includes proportional amplifier units, a multiplier unit, an adder unit, and integrator units. The proportional amplifier unit ① amplifies the output signal $u_{1}(t)$ of the integrator ⑤. The proportional amplifier unit ② amplifies the output signal ${\dot{u}}_{1}(t)$ of the integrator ④. The proportional amplifier unit ③ amplifies the output signal of the multiplier. The adder unit realizes the accumulation of the output signal of the proportional amplifier units. Since the adder has the function of an inverter at the same time, ${\ddot{u}}_{1}(t)$ can be acquired according to (42), and $u_{1}(t)$ can be obtained by the integral circuit ④ and the integral circuit ⑤. The $u_{1}(t)$ is input to the proportional amplifier circuit to form a closed loop; thus, a complete equivalent circuit is constructed. The equivalent circuit diagram of the silicon-micro resonant pressure sensor is shown in Figure 4. The black dotted boxes are the proportional amplifier modules, the orange dotted box is the multiplier, and the purple dotted box and red dotted box are the adder and integrator module, respectively, in Figure 4. Moreover, the parameters setting of each operational amplifier in the circuit is achieved by calculation using the geometric parameters of the resonant beam, according to the characterization model shown in Equation (24). This realizes the transformation between the dynamic model and verification circuit. Where $-(1/Q_{1})$ corresponds to U6A, −ε corresponds to U3A and U2A, the coefficient of output signal $u_{1}(t)$ corresponds to U4A and U5A, and the circuit parameters of the integrators U7A and U8A are designed by calculating the stiffness of the resonant beam. Therefore, the parameters characterization corresponding to the nonlinear vibration of the silicon-micro resonant pressure sensor is established by using the basic circuit units, and the equivalence between the verification circuit and the structural parameters of the silicon-micro resonant pressure sensor is proved. In order to obtain the output waveform with stable amplitude and frequency in a certain range, some parameters of the verification circuit have been optimized.

**Figure 5:**
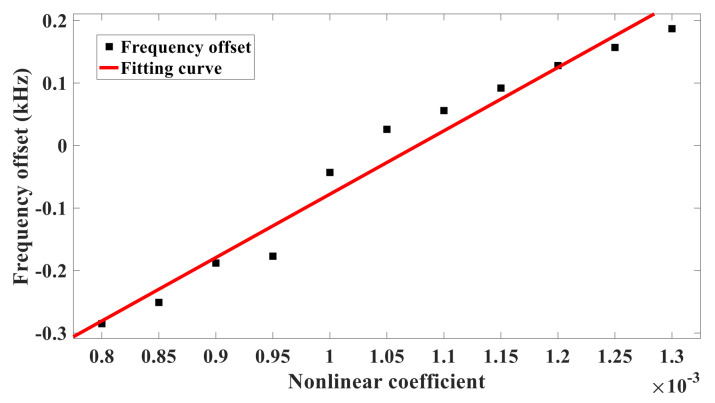
The frequency offset of the nonlinear coefficient.

**Figure 6:**
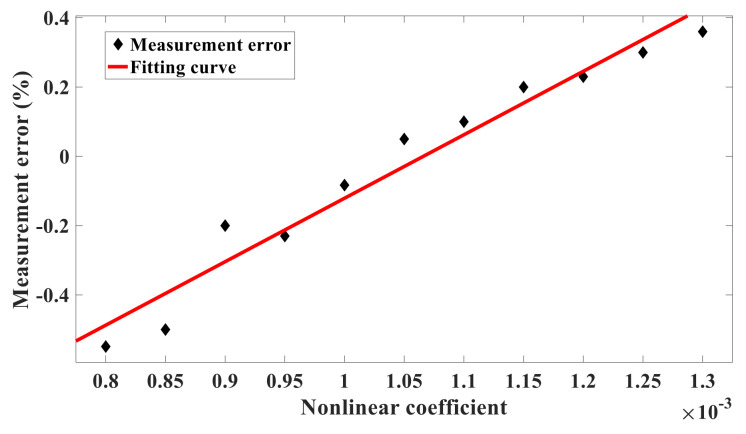
The influence of nonlinear coefficient on measurement error.

Since the nonlinear coefficient characterizes the strength of the nonlinear vibration, it is convenient to analyze the influence of nonlinear vibration on the measurement error of the silicon-micro resonant pressure sensor by changing the nonlinear coefficient. In Equation (42), the nonlinear term $u_{1}^{3}(t)$ is regulated by ε, while in the equivalent circuit, ε corresponds to U3A and U2A. Therefore, the nonlinear coefficient can be jointly determined by R10 and R12, Similarly, R18 and R20 can also regulate the output signal $u_{1}(t)$, thus keeping the resistance of R12 unchanged here, setting R10 with different resistances, and fine-tuning the feedback resistance R18 and the integrator capacitance C3 to make the output waveform stable. The frequency deviation can be obtained by the difference between the self-oscillation frequency of the output waveform and its natural frequency. The value of R10 and the corresponding frequency deviation are shown in Table 2. Figure 5 is the curve of frequency offset varying with nonlinear coefficient. The measurement error can be obtained from Equation (39). Figure 6 shows how the measurement error varies as the nonlinear coefficient does.

It can be seen from Figure 5 and Figure 6 that the frequency offset and measurement error are approximately linearly related to the nonlinear coefficient when the output waveform is stable. With the increase of the nonlinear coefficient, the frequency offset and measurement error also increase. According to (42), the measurement error of the silicon-micro resonant pressure sensor caused by nonlinear vibration is proportional to the strength of nonlinear vibration directly.

The experimental results of the circuit simulation confirm the influence of nonlinear vibration on the measurement error; moreover, they verify the reliability of the theoretical analysis conclusion given in the previous section.

## 4. The Measurement Error Compensation Method for Nonlinear Vibration

### 4.1. Compensation Principle

**Figure 7:**
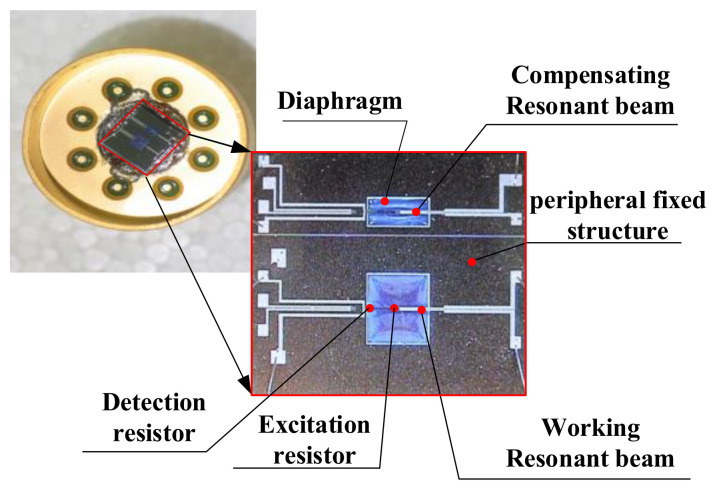
Double silicon-micro resonant pressure sensor.

As shown in Figure 7, the error compensation method for nonlinear vibration is introduced by taking the double beam silicon-micro resonant pressure sensor as an example. The sensitive structure consists of a square diaphragm, two resonant beams, and a peripheral fixed structure. One of the resonant beams is situated on the upper surface of the square diaphragm. When the diaphragm deforms under the measured pressure, the resonant beam is stretched and its natural frequency shifts, so it is called the working beam. Another resonant beam is in the fixed area, which isolates the influence of the measured. The natural frequency of this resonant beam is independent of the measured pressure. This resonant beam is mainly used to compensate for the natural frequency deviation of the working beam due to the nonlinear vibration, so it is called the compensation beam. Diffusion resistors are installed at the center and root of the upper surface of the working and compensation beam, respectively, which is used to excite and detect their vibration. All the parameters applied on the working and compensation beams are identical, such as the material, geometric dimension, excitation and detection resistance parameters, and the excitation signal, etc.

Under the measured pressure, the natural frequency of the working beam changes with the measured pressure, while the natural frequency of the compensation beam is independent of the measured pressure owing to the fixed structure. Because the parameters of the resonant beam are the same, when the resonant beam is in the nonlinear vibration due to the excessive amplitude, the natural frequency shift of the working beam caused by the nonlinear vibration is also approximately equal to the natural frequency offset of the compensation beam. Taking the difference between the natural frequency of the working beam and the compensation beam as the frequency output of the sensor can greatly reduce the sensor measurement error caused by the nonlinear vibration.

From (35), the resonant frequency of the resonant beam nonlinear vibration can be expressed as:

(43) $${\hat{\omega }}_{r}={\hat{\omega }}_{1}+\frac{3\hat{a}}{8r^{2}}{\hat{\omega }}_{1}\varepsilon$$

For both the working beam and compensation beam in the double silicon-micro resonant pressure sensor, the resonant frequency of them could be described in a normalized expression as:

(44) $${\hat{\omega }}_{ri}={\hat{\omega }}_{1i}+\frac{3\varepsilon {\hat{\omega }}_{1i}K_{1i}^{2}}{32\mu_{1}^{2}}={\hat{\omega }}_{1i}+\Delta {\hat{\omega }}_{ni}$$

where subscripts i=a, b represent the working beam and compensation beam, respectively, ${\hat{\omega }}_{1i}$ is the resonant beam natural frequency, and $\Delta {\hat{\omega }}_{ni}$ is the difference between the resonant frequency and the natural frequency of the resonant beam caused by the nonlinear vibration. The natural frequencies of the working beam and compensation beam can be written as follows according to (13):

(45) $${\hat{\omega }}_{1i}={\left[1+\frac{0.295l^{2}}{12EJ}N_{ri}+\frac{0.295l^{2}}{12EJ}N_{pi}+\frac{0.295l^{2}}{12EJ}N_{ti}\right]}^{0.5}\;\times \frac{4.73}{l^{2}}{\left(\frac{EJ}{\rho bh}\right)}^{0.5}$$

Since $-1<<0.295N_{ti}/(12EJ)<0$ and $0<<0.295N_{pi}/12EJ<<1$, (45) can be rewritten as:

(46) $${\hat{\omega }}_{1i}\approx {\hat{\omega }}_{i}+\Delta {\hat{\omega }}_{pi}+\Delta {\hat{\omega }}_{ti}$$

where

(47) $${\hat{\omega }}_{i}=\frac{4.73^{2}}{l^{2}}{\left(\frac{EJ}{\rho bh}\right)}^{0.5}{\left[1+\frac{0.295l^{2}}{12EJ}N_{ri}\right]}^{0.5}$$

(48) $$\Delta {\hat{\omega }}_{pi}=\frac{4.73^{2}}{24}{\left(\frac{EJ}{\rho bh}\right)}^{0.5}{\left[1+\frac{0.295l^{2}}{12EJ}N_{ri}\right]}^{0.5}\frac{0.295}{EJ}N_{pi}$$

(49) $$\Delta {\hat{\omega }}_{ti}=\frac{4.73^{2}}{24}{\left(\frac{EJ}{\rho bh}\right)}^{0.5}{\left[1+\frac{0.295l^{2}}{12EJ}N_{ri}\right]}^{0.5}\frac{0.295}{EJ}N_{ti}$$

where $\Delta {\hat{\omega }}_{pi}$ and $\Delta {\hat{\omega }}_{ti}$ are the changes of the resonant beam natural frequency caused by the measured pressure and the electrothermal excitation thermal effect, respectively. Substituting the Equation (47) into (45) yields:

(50) $${\hat{\omega }}_{ri}={\hat{\omega }}_{i}+\Delta {\hat{\omega }}_{pi}+\Delta {\hat{\omega }}_{ti}+\Delta {\hat{\omega }}_{ni}$$

The difference between the resonant frequency of the working beam and the compensation beam is:

(51) $${\overset{⌢}{\omega }}_{ra}-{\overset{⌢}{\omega }}_{rb}=({\overset{⌢}{\omega }}_{a}-{\overset{⌢}{\omega }}_{b})+(\Delta {\overset{⌢}{\omega }}_{pa}-\Delta {\overset{⌢}{\omega }}_{pb})+(\Delta {\overset{⌢}{\omega }}_{ta}-\Delta {\overset{⌢}{\omega }}_{tb})+(\Delta {\overset{⌢}{\omega }}_{na}-\Delta {\overset{⌢}{\omega }}_{nb})$$

As the geometric, material physical parameters and processing technology of the working beam and compensation beam are the same, there is ${\hat{\omega }}_{a}={\hat{\omega }}_{b}$. The compensation beam is in the isolation area, its natural frequency is not related to the measured pressure; as a result, the change of natural frequency caused by the measured pressure is $\Delta {\hat{\omega }}_{pb}=0$. The electro-thermal excitation parameters of the working beam and the compensating beam are the same. It can be considered that the change of the natural frequency of double beams caused by thermal effect is approximately equal, i.e., $\Delta {\hat{\omega }}_{ta}=\Delta {\hat{\omega }}_{tb}$.

**Figure 8:**
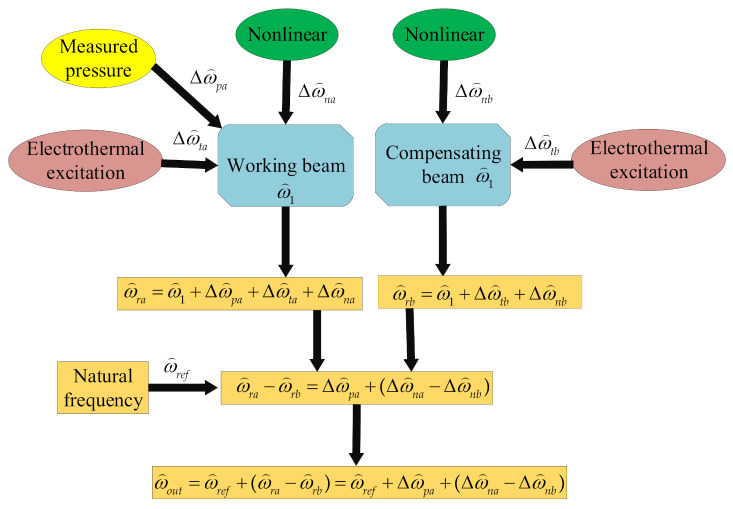
The block diagram of the error compensation system of the pressure sensor with the double resonant beam.

The frequency output of the double beam silicon-micro resonant pressure sensor is defined as:

(52) $${\hat{\omega }}_{out}={\hat{\omega }}_{ref}+\left({\hat{\omega }}_{ra}-{\hat{\omega }}_{rb}\right)={\hat{\omega }}_{ref}+{\hat{\omega }}_{pa}+\left(\Delta {\hat{\omega }}_{na}-\Delta {\hat{\omega }}_{nb}\right)$$

where ${\hat{\omega }}_{ref}$ is the natural frequency of the resonant beam under linear vibration without measured pressure. $\Delta {\hat{\omega }}_{na}-\Delta {\hat{\omega }}_{nb}$ is the frequency output of the double beam silicon-micro resonant pressure sensor caused by the nonlinear vibration. Based on the above calculation and analysis, as shown in Figure 8, the block diagram of the error compensation system of pressure sensor with double resonant beam is given.

The measurement error caused by the nonlinear vibration is given by:

(53) $$E_{double}=\frac{\Delta {\hat{\omega }}_{na}-\Delta {\hat{\omega }}_{nb}}{{\hat{\omega }}_{ref}+\Delta {\hat{\omega }}_{pa}}$$

**Figure 9:**
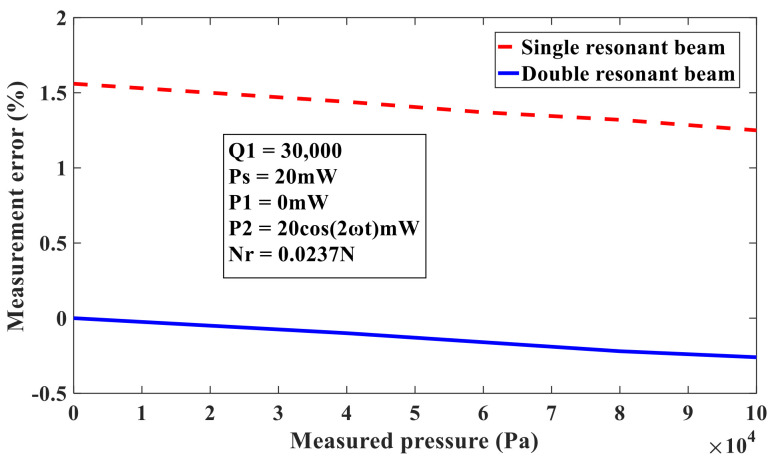
Measurement error of resonant pressure sensor caused by nonlinear vibration.

The measurement error (42) of the single beam resonant pressure sensor and caused by nonlinear vibration is compared with the measurement error (53) of the double beam silicon-micro resonant pressure sensor. This is shown in Figure 9, in which the quality factor $Q_{1}$ is 30,000, the static thermal power $P_{S}$ is 20mW, the dynamic thermal power components $P_{1}$ and $P_{2}$ are 0mW and 20cos(2ωt)mW, respectively, and the residual stress $N_{r}$ is 0.0237 N. When the measured pressure is zero, the axial internal force and amplitude of the working beam and compensation beam are the same, and the resonant frequency offset of the working beam and compensation beam caused by the nonlinear vibration is the same $\Delta {\hat{\omega }}_{na}=\Delta {\hat{\omega }}_{nb}$, so it can be seen from (53) that the measurement error of the double beam silicon-micro resonant pressure sensor caused by the nonlinear vibration without the measured pressure is zero. When the measured pressure increases gradually from zero, the bending coefficient of the working beam is less than the compensation beam, and the resonant frequency shift of the working beam caused by the nonlinear vibration is smaller than the resonant frequency shift of the compensation beam caused by the nonlinear vibration. The measurement error of the double beam silicon-micro resonant pressure sensor is negative and increases with the increase of the measured pressure. It also can be seen in Figure 9 that the measured pressure reaches 1 standard atmospheric pressure, and the measurement error of double beam silicon-micro resonant pressure sensor caused by nonlinear vibration is −0.26%, which is far less than that caused by the single beam resonant pressure sensor under the same working conditions.

### 4.2. Experiment of Error Compensation Method

**Figure 10:**
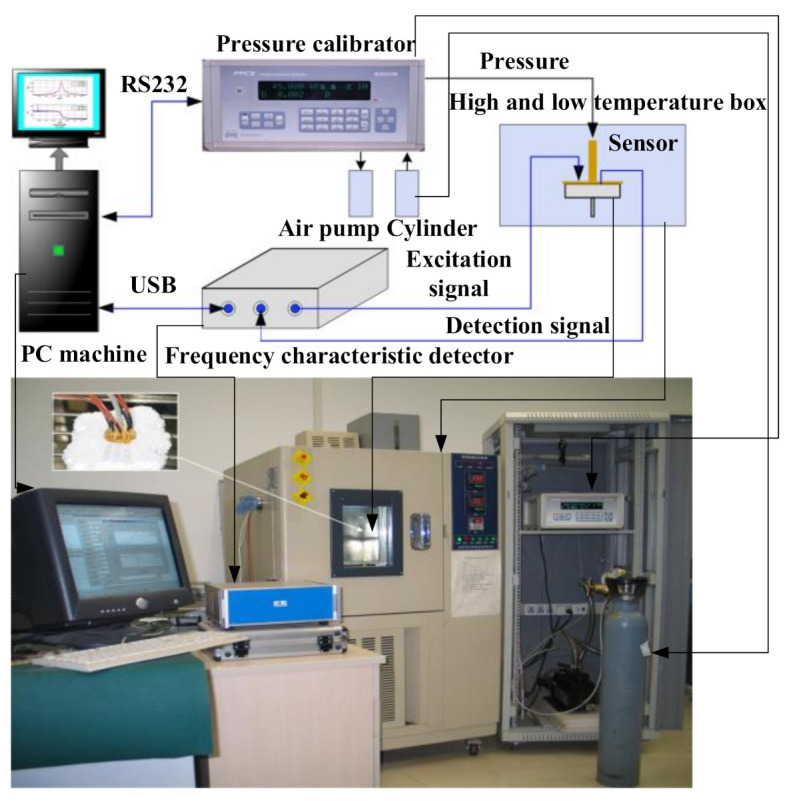
Nonlinear vibration frequency response test system.

In order to verify the feasibility of the nonlinear vibration error compensation method, the frequency response test system is used to obtain the nonlinear vibration frequency response curve of the double beam silicon-micro resonant pressure sensor under different static thermal power, measured pressure, and temperature, as shown in Figure 10.

It can be concluded that the frequency output offset caused by nonlinear vibration of the single beam resonant pressure sensor and double beam silicon-micro resonant pressure sensor are $\Delta {\hat{\omega }}_{na}$ and $\Delta {\hat{\omega }}_{na}-\Delta {\hat{\omega }}_{nb}$, respectively. In order to describe the effectiveness of the compensation method more intuitively, ξ is defined as the compensation rate of the nonlinear vibration error of the double beam silicon-micro resonant pressure sensor, and is formulated by:

(54) $$\xi =\left(1-\left|\frac{\Delta {\hat{\omega }}_{na}-\Delta {\hat{\omega }}_{nb}}{\Delta {\hat{\omega }}_{na}}\right|\right)\times 100\%$$

**Figure 11:**
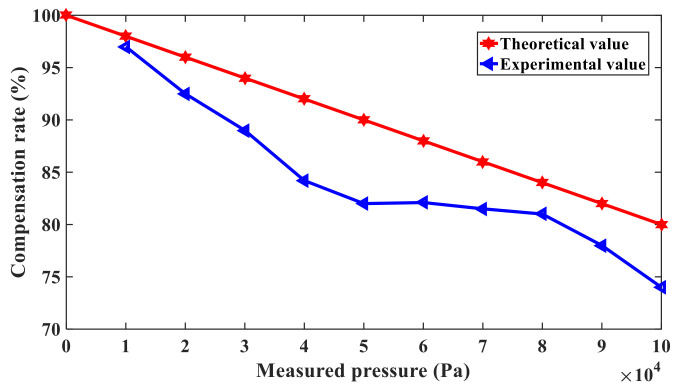
Compensation rate of resonant pressure sensor caused by nonlinear vibration.

If the measured pressure and excitation power were given, according to the nonlinear frequency response curve of the working beam and compensation beam, the resonant frequency shift caused by nonlinear vibration can be obtained, respectively. Then, the compensation rate of nonlinear vibration error is calculated by (54), given that the measured pressure is 10kPa, 20kPa, 30kPa, 40kPa, 50kPa, 60kPa, 70kPa, 80kPa, 90kPa, and 100kPa, and the excitation power and temperature are set to 98.70mW and 40 °C, respectively. The compensation rate changes with the measured pressure, as shown in Figure 11. The broken line and straight line represent the experimental and theoretical values of the nonlinear vibration compensation rate under different pressures, respectively. It is shown in Figure 11 that the differential compensation method can eliminate the nonlinear vibration error up to 97% at 0–1 standard atmospheric pressure. At the same time, the effectiveness of the compensation method is also proven.

**Figure 12:**
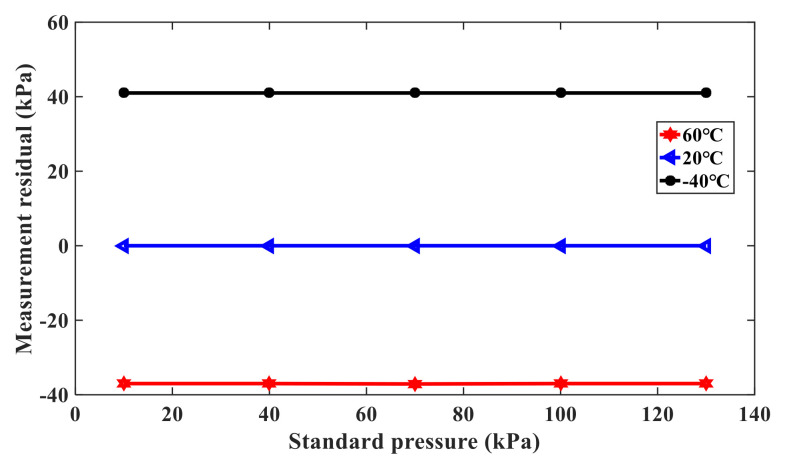
Measurement residual of the single resonant beam pressure sensor.

**Figure 13:**
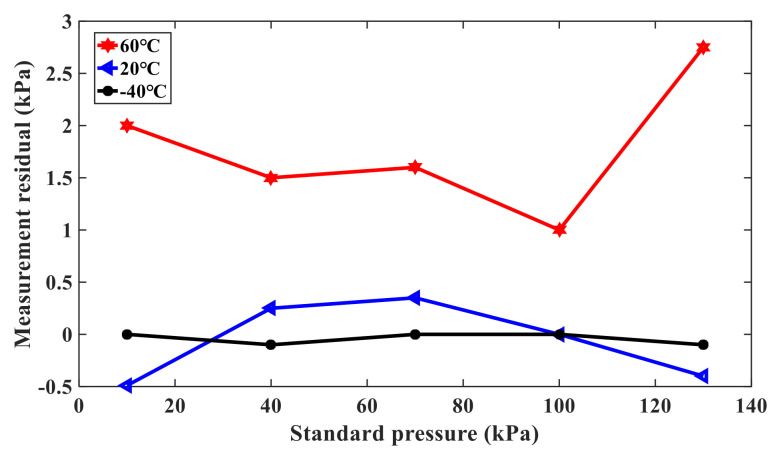
Measurement residual of the double resonant beam pressure sensor.

In order to further verify the advantages of the compensation method, the double resonant beam structure can be as a single resonant beam pressure sensor, except for the compensation beam. The output pressure can be calculated from the pressure characteristic curve of the sensor, and the measurement residual can be expressed as the difference between the output pressure and the standard input pressure. Figure 12 shows the variation of the measurement residual error with the single resonant beam pressure sensor, and Figure 13 shows the variation of the measurement residual error with the double resonant beam pressure sensors. When the measured standard pressure is 10kPa, 40kPa, 70kPa, 100kPa, and 130kPa, the excitation power is 98.70mW, the temperature is 20 °C, and the maximum measurement residual of the single resonant beam structure is 3.4kPa, while the maximum measurement residual of the double beam structure is 0.24kPa, which is about 1/10 of the former. It proves that the double resonant beam structure is more effective than the single resonant beam structure in nonlinear measurement error suppression. In addition, when the temperature is −40 °C, 20 °C, and 60 °C, respectively, the measurement residuals of the single resonant beam structure and double resonant beam structure pressure sensor are compared. It can be concluded that the measurement residual of the single resonant beam structure pressure sensor is smaller at 20 °C, but it is larger at −40 °C and 60 °C, and the minimum relative residual is 26%. At the same time, the results show that the maximum relative residual of the double resonant beam structure pressure sensor is 1.9% at −40 °C, 20 °C, and 60 °C, which further proves the effectiveness of the double resonant beam structure to compensate for the nonlinear error drift caused by temperature fluctuation.

Based on the above experiments results, it can be concluded that the compensation method of the double resonant beam structure is not sensitive to the temperature deviation. Additionally, the compensation rate is more significant in a low-pressure environment according to Figure 11, which has a promoting effect on the development of the aerospace industry.

## 5. Conclusions

- The working principle of the silicon-micro resonant sensor is analyzed, the model of the resonant beam is simplified, and the parameter characterization of the resonant beam nonlinear vibration is obtained. The multiscale method in the perturbation method is used to solve the parameter characterization to obtain the amplitude-frequency response and phase-frequency response. Furthermore, the measurement error of the resonant beam is obtained, and it is verified by the verification circuit that the frequency offset and measurement error are positively correlated with the nonlinear vibration.
- In order to compensate for the measurement error considered nonlinear vibration, a compensation method applying double silicon-micro resonant beams for the pressure sensor is proposed. The compensation principle, algorithms, and measurement error are discussed.
- A series of measurement experiments were carried out, which were used to obtain the nonlinear vibration frequency response curve under different measured pressure and temperature; it can be obtained that the measurement error and compensation rate were a good match in the predicted trends, which verifies the effectiveness of the compensation method.
- From the measured pressure residual experiments results, which further verified the advantages of the compensation method, it was also verified that the double resonant beam structure is not sensitive to the temperature deviation. Therefore, the double silicon-micro resonant beam pressure sensor has a promoting effect on the development of the aerospace industry.
- In the future, we will consider an algorithm that automatically tunes some of the parameters to counteract the nonlinearity induced frequency error instead of using the typical structural compensation approach.

## Figures and Tables

**Table 1:** Symbols and definitions.

Symbol	Definition
$\hat{w}$	Lateral vibration displacement
c	Viscous damping coefficient
E	Young’s modulus of materials
J	The moment of inertia of resonant beam cross section
ρ	Material density
v	Poisson’s ratio of materials
b	Width of the resonant beam
l	Length of resonant beam
h	Thickness of resonant beam
L	Edge length of the square diaphragm
$\alpha_{L}$	Coefficient of thermal expansion
ΔT	The average axial temperature rises of the resonant beam
$N_{n}$	The axial tension
$N_{t}$	The axial pressure caused by static thermal power
$N_{p}$	The axial tension caused by the measured pressure
$N_{r}$	The axial residual stress

**Table 2:** Resistance and frequency offset.

R10	Frequency Offset
80 Ω	−0.285 kHz
85 Ω	−0.251 kHz
90 Ω	−0.188 kHz
95 Ω	−0.177 kHz
100 Ω	−0.043 kHz
105 Ω	0.026 kHz
110 Ω	0.056 kHz
115 Ω	0.092 kHz
120 Ω	0.128 kHz
125 Ω	0.157 kHz
130 Ω	0.187 kHz

## Data Availability

The data are available upon request.
